# Analyzing Cell-free Genomic DNA in Spent Culture Media: Noninvasive Insight into the Blastocysts

**DOI:** 10.1055/s-0044-1788260

**Published:** 2024-07-23

**Authors:** Siddhartha Shankar Layek, Shrushti Kanani, Shilpa Doultani, Tejas Gohil, Sanket Patil, Ananthasayanam Sudhakar, Kathan Banubhai Raval, Karuppanasamy Kuppusamy, Sanjay Gorani, Sudharson Raj, Rafiya Sangameshwari, Himali Jadeja, Mini Mol P.

**Affiliations:** 1 National Dairy Development Board, Anand, Gujarat, India; 2 Department of Clinical Embryology, MGM School of Biomedical Sciences, MGM Institute of Health Sciences, Kamothe, Navi Mumbai, Maharashtra, India; 3 Department of Zoology, Gujarat University, Navrangpura, Ahmedabad, Gujarat, India; 4 Sabarmati Ashram Gaushala, Kheda, Gujarat, India; 5 Department of Anatomy, MGM Medical College, MGM Institute of Health Sciences, Kamothe, Navi Mumbai, Maharashtra, India

**Keywords:** single embryo culture, cf-DNA, spent media, noninvasive PGT, blastocysts

## Abstract

A commonly accepted standard protocol for noninvasive techniques for the genetic evaluation of an embryo remains elusive due to inconclusiveness regarding the volume of spent media to be acquired and the possibility of acquiring the same for subsequent analysis. Single embryo culture is imperative for standardizing noninvasive preimplantation testing using cell-free DNA (cf-DNA) released by individual developing embryos. This study aims to compare the development dynamics of single-drop embryonic culture against with group embryonic culture to establish a standardized protocol for noninvasive Preimplantation Genetic Testing (PGT) in bovine. A total of 239 cumulus–oocyte complexes were aspirated and subjected to in vitro maturation and fertilization. Among these, 120 embryos of day 3 were transferred to single-drop culture until the blastocyst stage. The single-drop culture drops were prepared using microdrops of 30 μL. At the blastocyst stage, spent media from all single-drop embryos were utilized for extracting cell-free genomic DNA to standardize the protocol. The blastocyst rate indicates no significant difference between the two culture methods, suggesting that single-drop culture is suitable for the process. Additionally, the extracted spent media yielded sufficient quantities of cf-DNA, supporting its potential use for PGT ( *p* < 0.05). These findings support the hypothesis that single-drop embryo culture is a viable method for cf-DNA extraction and confirm the potential of using DNA fragments from spent media as a reliable source for noninvasive PGT.

## Introduction

Infertility presents a significant challenge in modern medicine, recognized by the World Health Organization as a societal issue due to its widespread impact. In addressing infertility, various treatment approaches exist, including medication-based therapy, surgical interventions, and Assisted Reproductive Technology (ART). 1 ART has seen remarkable advancements since the birth of Louise Brown in 1978, with subsequent successes globally. 2

However, challenges persist, notably in genetic screening of embryos, where accurate detection of genetic defects is crucial for successful pregnancy outcomes. 3 Current techniques such as Preimplantation Genetic Testing (PGT) for Aneuploidy have shown effectiveness but raise concerns about embryo safety and procedural standardization. The emergence of noninvasive Preimplantation Genetic Testing (niPGT) offers a promising alternative, potentially reducing costs and improving outcomes. 3 Studies have shown niPGT's reliability in detecting genetic anomalies, with less risk of error than invasive techniques.

Innovations in ART also include noninvasive embryo monitoring through spent media analysis, which minimizes embryo manipulation and potential harm due to biopsy. 4 5 6 Additionally, research into group embryo culture highlights the importance of autocrine and paracrine communication in promoting embryonic development. 7 Despite advancements, questions remain regarding the suitability of single-drop culture for embryo development and also for getting sufficient sample volume to perform further procedures.

Furthermore, PGT has significant applications not only in human medicine but also in animal husbandry. In domestic animals, PGT is utilized to introduce desirable traits, enhance genetic diversity, and improve overall herd quality. 8 9 10 By selecting embryos with specific genetic characteristics, breeders can promote traits such as disease resistance, productivity, and longevity, contributing to the advancement of livestock breeding programs. 11 12 Selection of the embryos based on genomic evaluation would increase selection intensity and reduce generation interval leading to increased genetic gain. The comparison between single and group culture methods is essential to establish a process that maintains embryo production efficiency while providing noninvasive insights into the blastocysts. If single culture does not achieve embryo production efficiency comparable to group culture, it would not be adopted by laboratories focused primarily on producing embryos. Ensuring similar efficiency is crucial for the acceptance of this method for noninvasive embryo testing.

## Materials and Methods

### Materials

The study was conducted at the OPU-IVEP-ET laboratory of the National Dairy Development Board (NDDB), Anand. While bovine oocytes were sourced from NDDB, Anand, and frozen semen doses (FSDs) were obtained from Sabarmati Ashram Gaushala, Bidaj. Oocyte washing media, in vitro maturation (IVM), in vitro fertilization (IVF), in vitro culture (IVC) media, semen washing media, and density gradient media were sourced from VitroGen, Brazil. Standard equipment for embryo culture was utilized. DNA extraction was conducted using the QIAamp Circulating Nucleic Acid Kit from QIAGEN (catalog number/ID: 55114, Germany), while quantitative measurement of cf-DNA was performed using the Qubit 4 Fluorometer (Brand: Invitrogen™ Q33226, Fisher Scientific, Sweden) with the Qubit 1× dsDNA HS Assay Kit (catalog number: Q32851, Thermo Fisher Scientific, MA, United States).

### Methods

#### Sample Size and Cycle Number

A total of 14 ovum pickup (OPU) and in vitro embryo production cycles (OPU-IVEP) were conducted and a total of 239 oocytes were aspirated. Seven cycles were each subjected to single embryo culture and group embryo culture, respectively. Spent media were collected from each cycle involving single-drop embryo culture plates for DNA extraction and noninvasive Preimplantation Genetic Diagnosis protocol standardization.

The in vitro embryo production (IVEP) was carried out as per already standardized procedures in the laboratory with some minor modifications. 13 14 15 16 Brief details of the process are mentioned below in subsections “Ovum Pickup” to “In Vitro Culture.”

The grading of cumulus–oocyte complex (COC) and embryos was performed as per standards prescribed by the International Embryo Technology Society (IETS) in the Manual of the IETS, 5th Edition, to keep uniformity with the internationally followed norms.

#### Ovum Pickup

The OPU procedures were performed according to the standardized procedure in the laboratory for Transvaginal Ultrasound-guided OPU in cattle. Oocytes were aspirated using OPU media. All visible follicles were counted and aspirated using an OPU needle coupled to the aspiration line and a vacuum system.

#### In Vitro Maturation

**Fig. 1:**
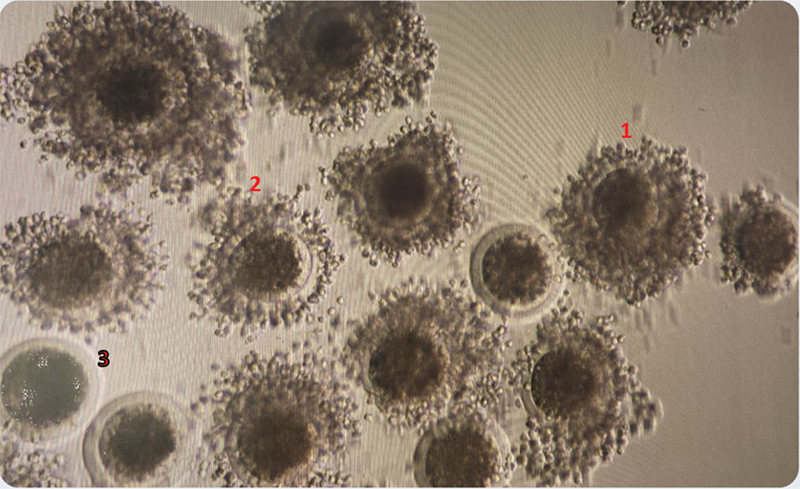
The figure illustrates various grading patterns of cumulus–oocyte complexes as outlined in the IETS Manual, 5th Edition. **Grade 1** : COCs have more than three complete and compact layers of cumulus cells covering the surface of the zona pellucida (ZP). The ooplasm is dense and has even granulation. **Grade 2:** COCs have one or two compact cumulus cell layers covering the surface of the zona pellucida. **Grade 3:** COCs have less than one complete layer of cumulus cells covering the surface of the ZP. COC, cumulus–oocyte complex; IETS, International Embryo Technology Society.

The oocytes were searched from the aspirated solution containing the COCs. Collected oocytes were graded according to IETS guidelines before processing for IVM. The grading was based on cumulus density and characteristics of ooplasm, viz., Grade 1 included COCs with more than three complete and compact layers of cumulus cells, Grade 2 had one or two compact cumulus cell layers, Grade 3 had less than one complete layer, and Grade 4 had expanded cumulus cell layers or signs of degeneration ( Fig. 1 ). The good-quality oocytes (Grades 1–3) were washed in IVM media and graded before placing them in preequilibrated IVM drop (media were preequilibrated overnight). Oocytes with maturation media were placed in an incubator with 5% CO _2_ in air (O _2_ concentration around 14–16%), 38.5 °C temperature, more than 90% relative humidity (RH), and incubated for around 22 hours for IVM.

#### In Vitro Fertilization

##### Oocyte Transfer

Oocytes were washed with IVF media and then transferred to preequilibrated IVF media drops (media were preequilibrated overnight) after approximately 22 hours of IVM.

##### Sperm Preparation and Fertilization

**Fig. 2:**
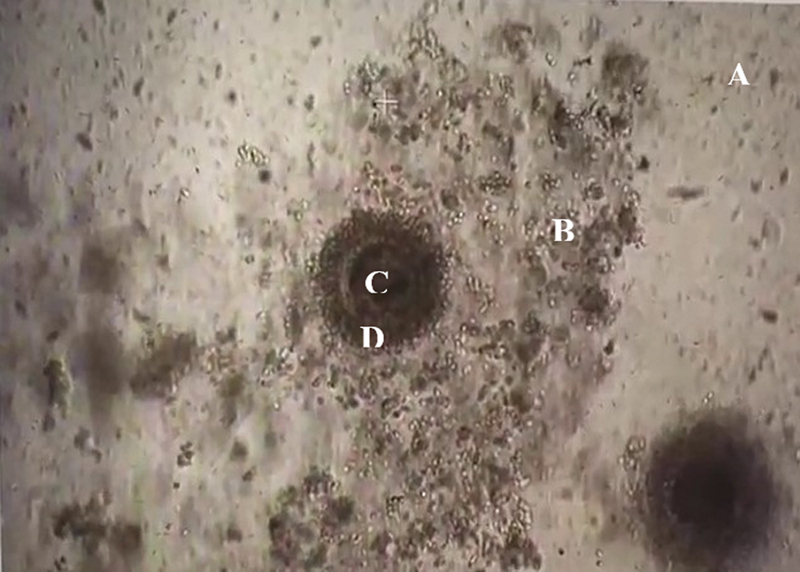
Oocyte with cumulus and sperm suspended in the media for fertilization (seen under an inverted microscope at 40 × ). (A) Sperm is suspended in the media for fertilization. (B) Cumulus cells. (C) Ooplasm. (D) Zona pellucida.

Preselected FSDs were used for fertilization. The semen underwent a two-step centrifugation process. Initially, the semen layered over the upper and lower gradient layers was centrifuged at 2,000 × g for 5 minutes. The pellet was then resuspended in IVF media and centrifuged again at 500 × g for 5 minutes. Finally, a predetermined volume of the sperm suspension was added to each fertilization drop. The Petri dish was then placed in an incubator with 5% CO _2_ in air (O _2_ concentration around 14–16%), 38.5 °C temperature, more than 90% RH, and incubated for around 18 hours for IVF ( Fig. 2 ).

#### In Vitro Culture

The presumptive zygotes (fertilized oocytes) underwent a denuding process following fertilization. This step involved carefully removing all surrounding cumulus cells using a denuding pipette. The denuding process was performed slowly and with utmost care to prevent damage to the zona pellucida. After complete denuding, the presumptive zygotes were washed sequentially in wash media and an IVC medium. Finally, the washed presumptive zygotes were transferred to preequilibrated IVC media drops (media were preequilibrated overnight) and placed back in a Mixed Gas benchtop incubator with 5% CO _2_ , 5% O _2_ , 90% N _2_ , 38.5 °C temperature, more than 90% RH for 7 days from the date of IVF.

#### Single-Drop Embryo Culture and Grading of Embryos

**Fig. 3:**
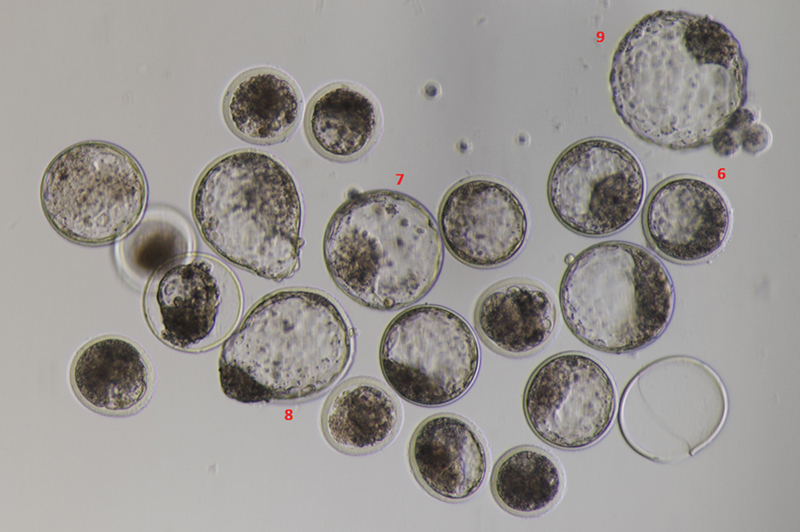
The figure presents the grading patterns of embryos on days 7 and 8 in accordance with the IETS Manual, 5th Edition. **Grade 6** : Blastocyst (days 7–8). **Grade 7:** Expanded blastocyst (days 8–9). **Grade 8** : Hatched blastocyst (day 9). **Grade 9:** Expanding hatched blastocyst (days 9–10). IETS, International Embryo Technology Society.

For single embryo culture, individual Day 3-grown embryos were transferred to separate single-drop embryo culture plates, where they were allowed to continue growing for the remaining days until Day 7. After 7 days from the IVF, the embryos were graded as per IETS guidelines, as outlined in the Manual of the IETS, 5th Edition. Embryos are assigned numerical codes from 1 to 9 based on their developmental stage, ranging from unfertilized or one-celled embryos on day 1 to expanding hatched blastocysts on days 9 and 10. For the current study, only Expanded blastocysts (day 7) and Blastocysts (day 7) were considered. Additionally, embryos undergo grading based on their quality, with numerical codes indicating their morphological integrity. Grade 1 represents excellent or good embryos, Grade 2 denotes fair quality, Grade 3 signifies poor quality, and Grade 4 indicates dead or degenerating embryos ( Fig. 3 ).

#### Spent Media Collection Protocol

On day 7 from IVF, blastocysts were graded, and spent media from single-drop cultures were collected. Spent media were labeled and stored for further analysis or immediate DNA extraction.

#### Isolation of Cell-free DNA

cf-DNA was extracted from spent media using the QIAamp Circulating Nucleic Acid Kit according to the manufacturer's instructions. The extraction process involved various steps including lysate preparation, column purification, and elution.

#### Quantification of Cell-free DNA

Quantification of cfDNA was performed using the Qubit fluorometer 4.0 in combination with the Qubit 1× dsDNA HS Assay Kit. Samples were diluted in Qubit working solution before quantification as per the manufacturer's instructions.

## Results

### Phase 1: Embryo Culture Efficiency

#### Comparison of Group versus Single Embryo Culture

A comparative analysis between group embryo culture and single-drop embryo culture methods revealed comparable trends in embryo formation efficiency across various cycles. Both methods exhibited variability in blastocyst rates, with single-drop embryo culture demonstrating a higher average blastocyst rate (40.6%) compared to group embryo culture (27.5%). However, statistical analysis indicated no significant difference between the two culture methods ( *p* = 0.09). The findings suggest that while single-drop embryo culture may offer slightly higher efficiency, both methods are suitable for supporting blastocyst development in IVEP cycles ( Table 1 ; Figs. 1 2 3 4 ).

**Fig. 4:**
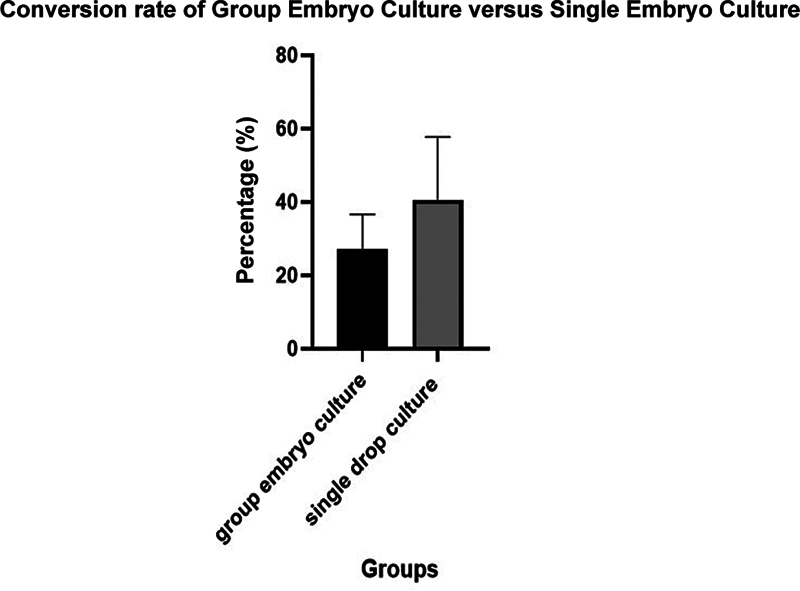
Biostatistics graph with an error bar of group embryo culture versus single embryo culture. The conversion rate of group embryo culture versus single embryo culture; data are expressed as mean ± SEM ( *n* = 7) 13.58 ± 7.410 when compared between the group embryo culture samples and single embryo culture ( *p* -value = 0.09), which shows there is no significant difference between the two culture groups; therefore, the Null Hypothesis is accepted.

**Table 1:** Comparison of group embryo culture versus single embryo culture

Cycle number	Group embryo culture			Single-drop embryo culture		
	Number of oocytes kept in in vitro culture	Number of embryos formed	Conversion rate	Number of oocytes kept in in vitro culture	Number of embryos formed	Conversion rate
1	10	3	30%	8	2	25%
2	24	8	33%	12	4	33.33%
3	6	1	17%	25	10	40%
4	13	5	38.46%	9	7	77.78%
5	25	9	36%	25	10	40%
6	17	3	17.65%	17	6	35.29%
7	24	5	20.83%	24	8	33%

### Phase 2: Cell free-DNA Release by Single Embryos

**Fig. 5:**
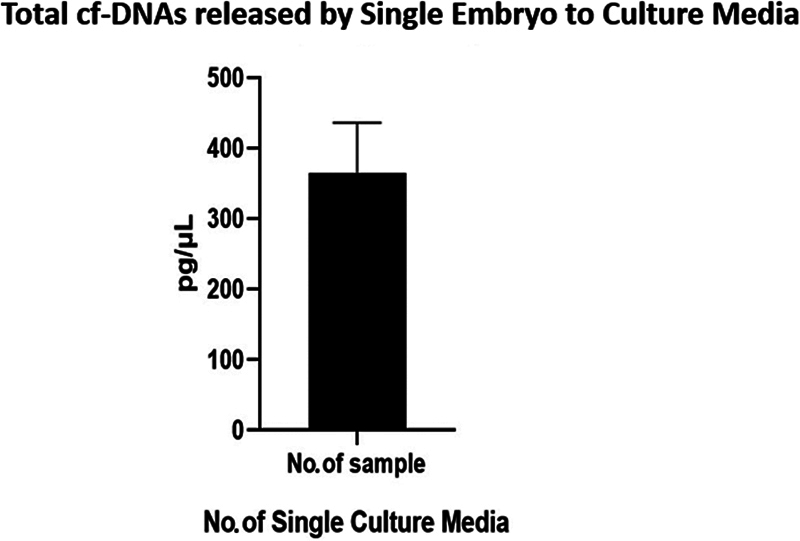
Biostatistics graph with an error bar of total cf-DNA released by a single embryo to culture media. Total cf-DNA released by a single embryo to culture media (graph a); data are expressed as mean ± SEM ( *n* = 48) *p* < 0.0001, 365 ± 10.25 between the samples. As there is a significant difference, the Null hypothesis is rejected and it validates that DNA fragments retrieved from spent media can be used for the noninvasive method. cf-DNA, cell-free DNA.

In the second phase, the release of cell-free DNA (cf-DNA) by single embryos into culture media was investigated. A total of 48 samples were analyzed across seven cycles. The mean quantity of cf-DNA released by single embryos was found to be 365 ± 10.25 pg/μL. Statistical analysis revealed a significant difference in cf-DNA release between samples ( *p* < 0.0001), validating the potential use of DNA fragments retrieved from spent media for noninvasive genetic analysis ( Fig. 5 ).

Figures 3 and 5 visually represent the data and statistical analyses conducted in this study, providing insights into the efficiency of embryo culture methods and the release of cf-DNA by single embryos.

## Discussion

Developing a noninvasive method for the evaluation of genetic insight into an embryo is always desirable considering the damaging effects of the biopsy process. Further, zona-damaged embryos are not preferred for the international trade of bovine embryos considering the potential to transmit diseases between boundaries. Thus, it is imperative to develop and optimize a single embryo culture method for developing an alternative, and without a single embryo culture, it is not possible to get insight into a particular embryo. The comparable result of single embryo culture compared to group culture may be explained by the fact that the sole nutrition from the culture media is devoted to a single embryo. 17 Aneuploid embryos in IVF, which can range from 20 to 100%, present significant biological challenges. 18 These chromosomal abnormalities commonly result from errors in chromosome segregation during meiosis, 19 influenced by factors such as advanced parental ages, eating habits, and lifestyle. Such aberrations significantly contribute to early pregnancy miscarriages and severe chromosomal disorders. Research indicates that the outcomes of ART procedures can be improved through PGT for aneuploidy, which facilitates the selection of embryos with optimal chromosomal integrity. 19

The global use of PGT is on the rise, typically involving invasive methods like polar body, blastomere, trophectoderm biopsy, or blastocentesis, to extract embryonic DNA. 20 However, these methods come with technical constraints and ethical considerations. Two primary issues spark controversy within the scientific community: the need for specialized instrumentation and skilled practitioners to ensure embryo viability, and concerns about potential harm to the embryo shared by both medical professionals and patients. 18 Recently, the use of spent culture media (SCM) as an alternative source of embryonic DNA has been proposed. Research has documented the presence of cf-DNA in SCM, highlighting its potential for niPGT to evaluate the genetic characteristics of IVF-derived preimplantation human embryos. 20 Despite this, a standardized noninvasive protocol remains elusive due to the availability of low sample volume at the start. 21 22 23 The widespread use of group embryo culture media in IVF clinics further complicates this, as single embryo culture is crucial for getting spent media specific to each embryo. 24

Numerous studies have yielded conflicting findings on the efficacy of group versus single embryo culture. To address these discrepancies and establish a standardized noninvasive protocol, the current study used bovine oocytes, which have similar germline-specific molecular profiles to humans, providing a relevant comparative framework. 20 According to Brouillet et al, 20 SCM can be collected at various preimplantation developmental stages, including cleaved embryos with fewer than six cells on day 3 and early blastocysts. This provides further benefits to invasive PGT (iPGT), which relies heavily on embryo biopsy or blastocentesis. SCM-based PGT offers a viable option for assessing cultured embryos with diminished implantation potential, 26 which are not suitable for iPGT. SCM-PGT features a rapid turnaround time (less than 12 h from SCM collection to genetic analysis), potentially providing results before embryo transfer or cryopreservation. 19 27

The amount of cf-DNA released by embryos varies significantly, impacting ART. Currently, embryo viability assessment often relies on invasive procedures like embryo biopsy or PGT, which can be costly, time-consuming, and risky. This underscores the need for further research to fully understand cf-DNA release by embryos and to optimize its use in noninvasive embryo assessment. 28 29

## Conclusion

In conclusion, the findings of this research highlight the promising potential of single-drop embryo culture in enhancing the efficiency of noninvasive preimplantation testing. The observed superiority in blastocyst quality and higher mean conversion rate suggest that single-drop culture may offer a more effective approach for selecting embryos with optimal genetic integrity, particularly in cases where genetic disorders or mutations are of concern. Additionally, the variability in cf-DNA release underscores the importance of further research to fully understand its implications and optimize its use in noninvasive embryo assessment. Overall, these findings contribute to the growing body of evidence supporting the adoption of single-drop culture as a preferred method for noninvasive preimplantation testing in IVEP.
